# Neurosyphilis complicated with pial arteriovenous fistula: A rare case report

**DOI:** 10.1097/MD.0000000000017770

**Published:** 2019-11-11

**Authors:** Lingmei Xu, Fugang Han

**Affiliations:** Department of Radiology, Affiliated Hospital of Southwest Medical University, 25 Taiping Road, Luzhou, Sichuan, China.

**Keywords:** magnetic resonance imaging, neurosyphilis, pial arteriovenous fistula

## Abstract

**Introduction:**

Neurosyphilis is a chronic, infectious disease of the central nervous system. Pial arteriovenous fistulae (PAVF) are rare vascular malformations. Both can cause vascular damage, but it is quite rare for both to present at the same time.

**Patient concerns:**

Here we present a 66-year-old man with affective disorder, hypomnesia, and recent convulsions. Magnetic resonance imaging revealed cerebral swelling, hyperintensity in the cortex/subcortex, and multiple lacunar cerebral infarctions. Computed tomography angiography revealed the presence of a pial arteriovenous fistula.

**Diagnoses:**

Based on laboratory tests and imaging, diagnoses of neurosyphilis and pial arteriovenous fistula were made.

**Interventions:**

Antisyphilis therapy was provided.

**Outcomes:**

Symptoms improved and antisyphilis treatment continued as an outpatient. No intracranial hemorrhage was seen 6 months later.

**Conclusion:**

*Treponema pallidum* infection may be related to the formation of PAVF, and may also promote the progression of it; however, further work is required to confirm this.

## 1 Introduction

Neurosyphilis is a chronic central nervous system (CNS) disease caused by the bacteria, *Treponema pallidum*. Although the incidence of syphilis is not as high as other sexually transmitted diseases, there are still a large number of cases noted worldwide. For example, in 2012, there were an estimated 5.6 million new cases globally, with many of these in lower, or lower- to middle-income economies.^[1]^ What makes this case so striking is the rarity of neurosyphilis coexisting with pial arteriovenous fistulae (PAVF). In this report, we present a patient with both neurosyphilis and PAVF. To the best of our knowledge, there has been no such case reported previously. Written informed consent was acquired for the publication of this case report.

## 2 Case report

A 66-year-old male was admitted to a neurology department with a 6-month history of affective disorder, hypomnesia, and convulsions. A physical examination revealed a twitching muscle on the left hand side of his face, his mouth tilted to the left, and a weakness in the muscles of the left lower limb. He had a history of brain atrophy and multiple lacunar infarctions, which improved with medicine (details unknown).

**Figure 1:**
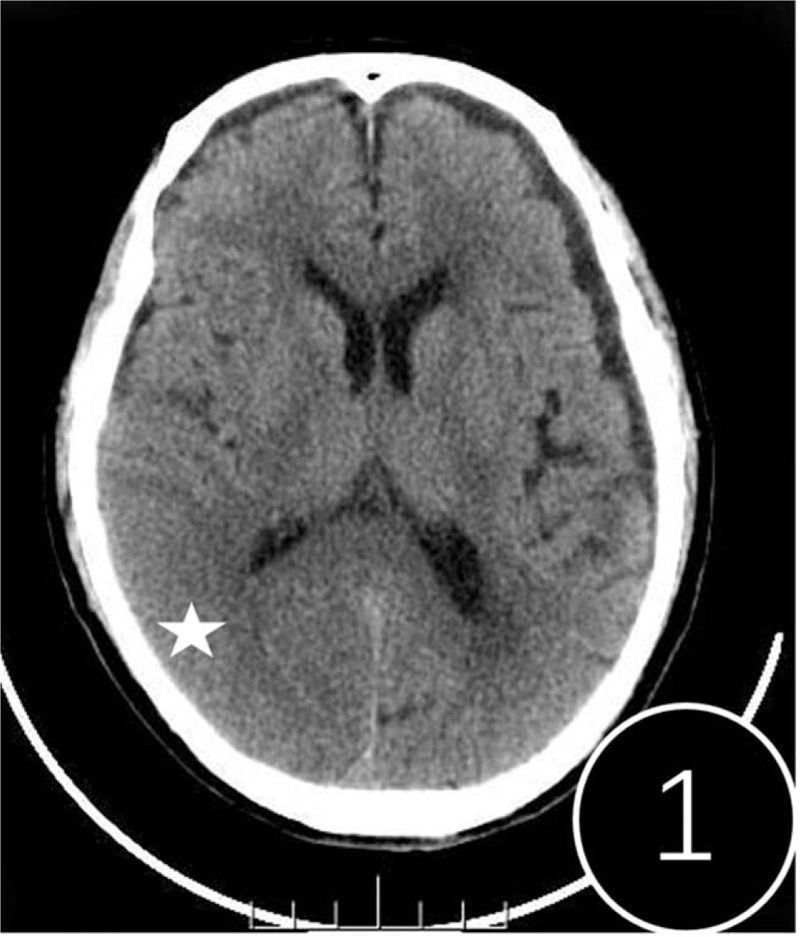
Brain computed tomography showed that the volume of the right temporal occipital lobe (pentacle) increased and the sulcus in the corresponding area became shallow and disappeared.

**Figure 2:**
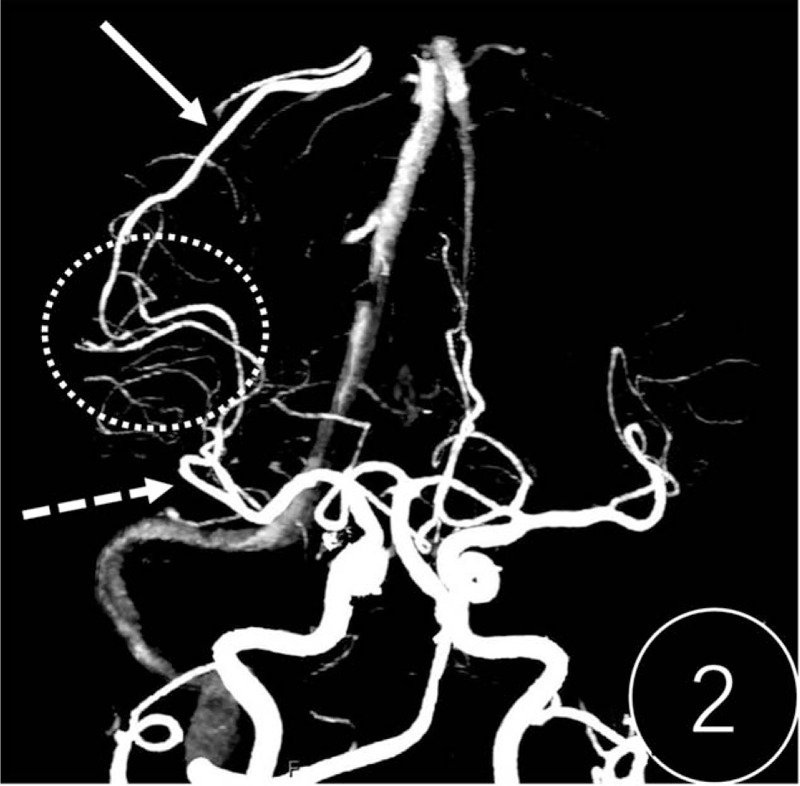
Computed tomography angiography revealed that the feeding artery of the pial arteriovenous fistula (PAVF) was one of the branches of the right middle cerebral artery (virtual arrow). The draining vein (solid arrow) thickened and converged into the superior sagittal sinus. The feeding artery communicated directly with the draining vein (circle area).

**Figure 3:**
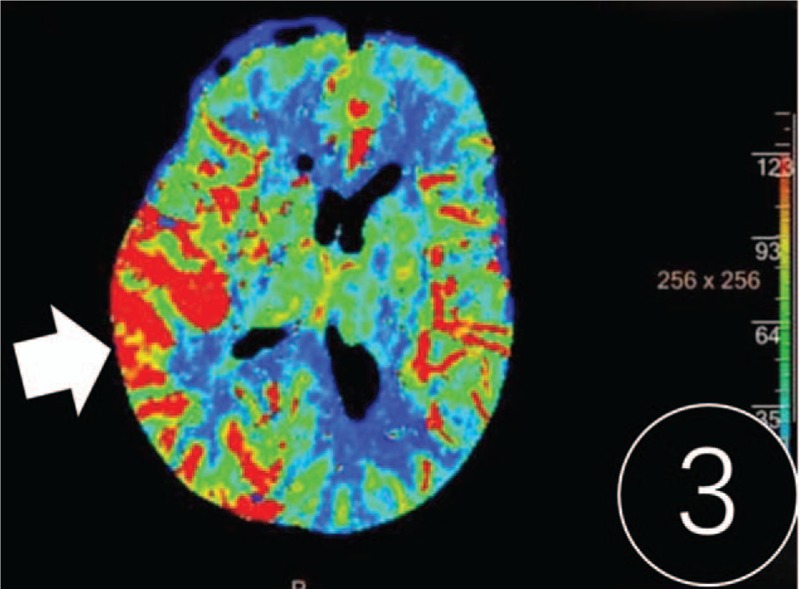
Computed tomography perfusion imaging reflected that the cerebal blood volume of the right temporal occipital lobe was significantly higher than that of the contralateral side.

**Figure 4:**
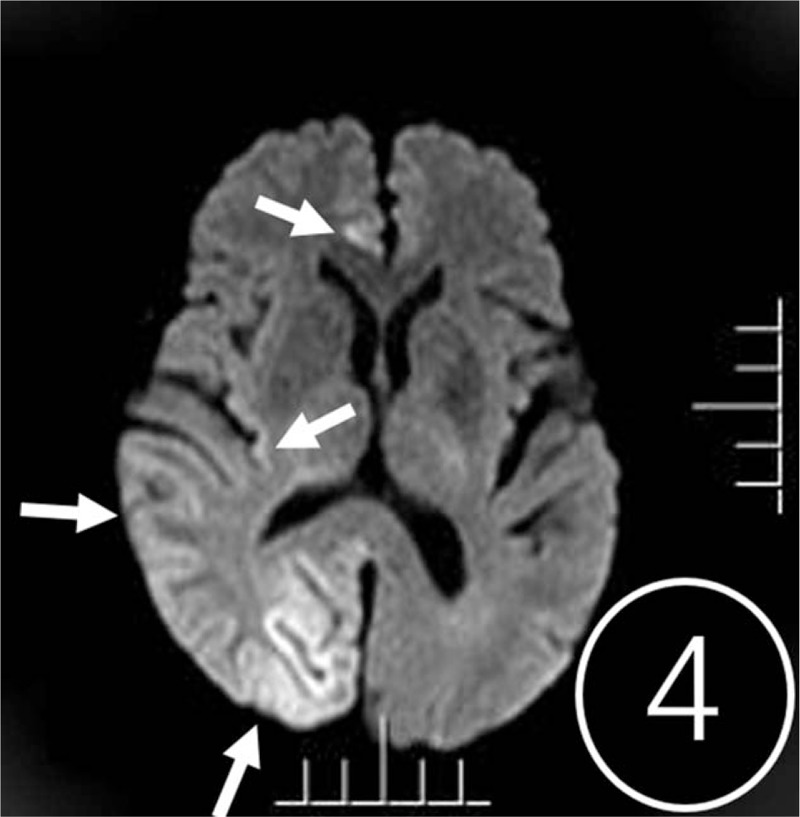
Diffusion-weighted imaging (DWI) showed multiple regions of the cortical/subcortex displayed hyperintensity in the right hemisphere (arrow).

A computed tomography (CT) scan revealed cerebral swelling in the right temporal occipital lobe, but no hemorrhage (Fig. 1). T2-weighted, fluid-attenuated inversion recovery (FLAIR) and diffusion-weighted (DWI) magnetic resonance imaging showed band-like hyperintense signaling in the cortex/subcortex of the right hemisphere (Fig. 2). Multiple patchy abnormal signals were seen in the centrum semiovale and periventricular regions (hyperintense signals on T2 and FLAIR, hypointense signals on DWI). Subsequent CT perfusion imaging revealed that perfusion of the right hemisphere was significantly higher than the contralateral hemisphere (Fig. 3). CT angiography revealed a PAVF in the same region (Fig. 4). A lumbar puncture revealed high levels of protein (1.347 g/L), and an elevated leukocyte count (23 × 10^6^cells/L) in the cerebrospinal fluid (CSF). *T pallidum* hemagglutination assay for syphilis was positive in the patient's blood and CSF.

Based on the clinical presentation, and results from subsequent neuroimaging and laboratory tests, a diagnosis of neurosyphilis with coexisting PAVF was made, and high-dose penicillin was administered. To prevent a Jarisch-Herxheimer reaction, dexamethasone was administered simultaneously. Before that, treatments for symptoms, such as diazepam, were used for convulsions. After subsequent 1 week of antibiotic treatment, the frequency of convulsions decreased significantly, the muscle strength was also beginning to recover. The patient requested outpatient treatment and refused additional testing. During the next 6 months of telephone follow-up, there was no intracranial hemorrhage, the most common complications of PAVF.

## 3 Discussion

Neurosyphilis may present at any stage following syphilis infection. It is thought to occur because of the invasion of the CNS by *T pallidum*, and the associated immune response. Invasion of the CNS by *T pallidum* can lead to lesions in the meninges, cerebrovasculature, and spinal cord. The invasion of the meninges results in the infiltration of lymphocytes, as part of the immune response. Invasion of the larger blood vessels leads to necrosis of the tunica media and proliferation of the tunica intima of the vessel walls. These are common characteristics of endarteritis obliterans of the medium and large vessels, and are considered to be typical lesions seen in neurosyphilis. Invasion of brain parenchyma leads to disruption of the architecture of the cerebral cortex, neuronal loss, and proliferation of astrocytes and microglia.^[2]^ Because of the extensive invasiveness of *T pallidum*, the clinical manifestations of neurosyphilis are diverse, and the symptoms are nonspecific, consisting of many symptoms and signs also seen in other neurological disorders.^[3]^

Many signs of neurosyphilis can be detected by imaging, owing to its complicated pathophysiology. Meningeal involvement may present as leptomeningeal thickening, leptomeningeal enhancement, and signal abnormalities. Signs of vascular involvement can be seen directly, such as stenosis and occlusion, and indirectly, such as cerebral infarction. Involvement of the brain parenchyma can manifest as brain atrophy, brain swelling, cerebral infarction, white matter lesions,^[4]^ and cerebral syphilitic gumma.^[5]^ This case presented with cerebral infarction, brain swelling, multiple cortical/subcortical regions of band-like hyperintensity, increased perfusion of the right hemisphere, and PAVF. Each of these findings could have arisen from another source; however, these were deemed unlikely because of a positive hemagglutination assay, confirming syphilis infection.

PAVF is a rare vascular malformation that differs from arteriovenous malformations (AVM) and from dural arteriovenous fistulas (DAVFs). PAVF is characterized by an arterial feeding vessel from pial or cortical arteries, and a large, lobulated venous varix or aneurysm. Where they differ from AVM is that PAVFs lack a true nidus. They differ from DAVF in that DAVF are fed by branches of meningeal arteries, and are located in the dura, rather than in the brain parenchyma, which is where PAVFs are found. The pathophysiological mechanism of PAVF is still unclear. It can result from trauma, previous surgery, or may be congenital.^[6]^ Embryonic cerebrovascular dysplasia may also be related to the formation of PAVF.^[7]^ In addition, it can also appear in patients as part of Rendu-Osler-Weber or Ehler Danlos syndromes.^[8]^ Our patient had no history of craniocerebral trauma or surgery, and no family history or clinical features of Rendu-Osler-Weber, or Ehler Danlos, syndrome. However, this patient was also diagnosed with neurosyphilis, so it is possible that the blood vessels became infected with *T pallidum*, causing vascular wall damage. Therefore, we speculated that *T pallidum* may be have been related to the formation of PAVF.

The pathogenesis of PAVF is unclear because of its rarity. By definition, there are no capillaries between the feeding artery and the draining vein of PAVF. The absence of capillary channels permits a low resistance to blood flow from the artery to the vein.^[9]^ This abnormal flow of blood results in progressive dilatation of both the feeding artery and the draining vein. Over time, abnormal hemodynamic stresses on the walls of the blood vessels may cause chronic endothelial alterations, leading to the destruction of the internal elastic lamina, increased endothelial permeability, and proliferation in the tunica intima.^[10]^ Therefore, with the development of PAVF, other adverse consequences may have occurred, such as vasogenic edema^[10]^ or hemorrhage.^[11,12]^ Around the PAVF, there was no definite hemorrhage seen in the CT scan, but vasogenic edema was found in the DWI of our patient. It is difficult, however, to determine whether this edema was caused by neurosyphilis or PAVF.

When reviewing the literature, it is clear that conservative treatment of PAVF is associated with a poor prognosis. Nevertheless, in our patient, *T pallidum* may have also caused damage to the vascular wall, which could have accelerated the progression of PAVF. Previous studies have shown that PAVF can be successfully treated through closure of the shunt by endovascular^[13,14]^ or surgical means.^[15]^ Unfortunately, the patient chose to leave the hospital and refused further imaging, in spite of the fact that our team strongly emphasized the seriousness of the disease to him.

In summary, this report shows that an additional complication of neurosyphilis may be PAVF, which has not previously been reported in the literature. It is important to note when analyzing images of brain edema, infectious lesions may not be the only cause, and that vascular abnormalities may also exist. The information gathered through this report is limited because of the poor adherence of the patient, and also because of a lack of follow-up after discharge. In light of this, we don’t know the exact course or final outcome of the patient.

## 4 Conclusion

Neurosyphilis complicated with PAVF is quite rare. Our observations of concurrent disease suggest that there may be a link between the two; however, further investigation would be required to assess the accuracy of this theory.

## Acknowledgment

The authors thank Editage (www.editage.com) for English language editing.

## Author contributions

**Conceptualization:** Fugang Han.

**Data curation:** Lingmei Xu.

**Formal analysis:** Lingmei Xu.

**Investigation:** Lingmei Xu.

**Methodology:** Lingmei Xu.

**Project administration:** Lingmei Xu.

**Resources:** Fugang Han.

**Software:** Lingmei Xu.

**Supervision:** Fugang Han.

**Validation:** Lingmei Xu, Fugang Han.

**Visualization:** Lingmei Xu.

**Writing – original draft:** Lingmei Xu.

**Writing – review & editing:** Fugang Han.

Fugang Han orcid: 0000-0001-6191-5077.

## References

[1] Newman L Rowley J Vander Hoorn S Global estimates of the prevalence and incidence of four curable sexually transmitted infections in 2012 based on systematic review and global reporting. PLoS One 2015;10:e0143304. 26646541 10.1371/journal.pone.0143304 PMC4672879

[2] Berger JR Dean D Neurosyphilis. Handb Clin Neurol 2014;121:1461–72. 24365430 10.1016/B978-0-7020-4088-7.00098-5

[3] Marra CM Neurosyphilis. Continuum (Minneap Minn) 2015;21:1714–28. 26633785 10.1212/CON.0000000000000250

[4] Faraut E Wallon D Gueit E MRI findings in a case of Lissauer form of neurosyphilis. Acta Neurol Belg 2018;118:113–4. 28939990 10.1007/s13760-017-0833-4

[5] Shao X Di Qiang Liu Y Diagnosis and treatment of cerebral syphilitic gumma: a report of three cases. Front Neurosci 2018;12:100. 29535598 10.3389/fnins.2018.00100 PMC5835125

[6] Xin SB Wang GB Liu WJ Congenital intracerebral pial arteriovenous fistula: a case report. J Neurol Surg A Cent Eur Neurosurg 2018;79:173–6. 29241267 10.1055/s-0037-1608842

[7] Hoh BL Putman CM Budzik RF Surgical and endovascular flow disconnection of intracranial pial single-channel arteriovenous fistulae. Neurosurgery 2001;49:1351–64. 11846934 10.1097/00006123-200112000-00011

[8] Campos C Piske R Nunes J Single hole high hlow arteriovenous fistula a characteristic presentation of Rendu-Osler–Weber disease in a young adult treated by endovascular approach case report. Int Neuroradiol 2002;8:55–60. 10.1177/159101990200800110 PMC3572524 20594513

[9] Goel A Jain S Shah A Pial arteriovenous fistula: a brief review and report of 14 surgically treated cases. World Neurosurg 2018;110:e873–81. 29191547 10.1016/j.wneu.2017.11.121

[10] Masuoka J Sakata S Maeda K Intracranial pial single-channel arteriovenous fistula presenting with significant brain edema. J Neurosurg 2008;109:497–501. 18759583 10.3171/JNS/2008/109/9/0497

[11] Kim HM Cho JH Kim KH Onyx embolization of intracranial pial arteriovenous fistula. J Cerebrovasc Endovasc Neurosurg 2016;18:291–5. 27847777 10.7461/jcen.2016.18.3.291 PMC5104858

[12] Lee JS Oh CW Bang JS Intracranial pial arteriovenous fistula presenting with hemorrhage: a case report. J Cerebrovasc Endovasc Neurosurg 2012;14:305–8. 23346547 10.7461/jcen.2012.14.4.305 PMC3543917

[13] Zenteno M Lee A Satyarthee GD Endovascular management of intracranial pial arteriovenous fistulas: experience of largest series at a single center over six years. J Neurosci Rural Pract 2018;9:406–9. 30069100 10.4103/jnrp.jnrp_455_17 PMC6050784

[14] Ye M Zhang P Transarterial balloon-assisted glue embolization of pial arteriovenous fistulas. World Neurosurg 2018;115:e761–7. 29729454 10.1016/j.wneu.2018.04.171

[15] Da Silva Martins WC de Albuquerque LAF de Souza Filho CBA CBA Surgical treatment of the intracranial pial arteriovenous fistula. Surg Neurol Int 2015;6:102. 26110083 10.4103/2152-7806.158518 PMC4476140

